# Novel Uses for Lipid-Lowering Agents

**DOI:** 10.6004/jadpro.2016.7.2.4

**Published:** 2016-03-01

**Authors:** Megan Brafford May, Ashley Glode

**Affiliations:** Baptist Health Lexington, Lexington, Kentucky; University of Colorado Skaggs School of Pharmacy and Pharmaceutical Sciences, University of Colorado Anschutz Medical Campus, Aurora, Colorado

## Abstract

Statin use leads to a reduction in the downstream products of the mevalonate pathway. Knowledge of this pathway has led scientists to investigate the role of statins in cancer prevention and treatment. Statins appear to possess a variety of pleiotropic effects, including inhibition of cell proliferation; enhanced apoptosis; and modulation of inflammation, endothelial function, and angiogenesis. In cancer specifically, experimental studies have found that statins may induce cancer cell apoptosis and inhibit tumor growth, angiogenesis, and metastasis. These mechanisms have steered researchers into evaluating the possible benefit of statins in the prevention and treatment of malignancies. This review will discuss the literature supporting the use of statins to prevent and treat cancer.

The HMG-CoA (3-hydroxy-3-methylglutaryl-coenzyme A) reductase inhibitors, more commonly referred to as statins, have historically been used for their ability to improve lipid profiles and reduce cardiovascular morbidity and mortality. Approximately 11% of the US population is prescribed statins, with prescription rates increasing to 44% in those older than 65 due to a recent change in statin therapy guidelines (Lochhead & Chan, 2013). Statins mimic the natural substrate, HMG-CoA, and compete for binding to the HMG-CoA reductase (HMGCR) enzyme, which catalyzes the conversion of HMG-CoA to mevalonate. The conversion to mevalonate is an early and rate-limiting step in the biosynthesis of cholesterol. Statins increase the rate of removal of cholesterol from the body and reduce cholesterol production by arresting the conversion of HMG-CoA to mevalonate (refer to Figure).

**Figure:**
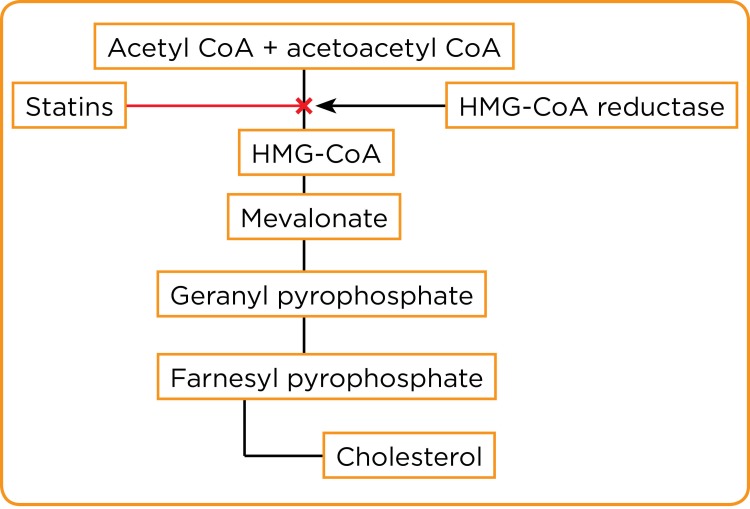
Cholesterol biosynthesis pathway.

The reduction of downstream products in the mevalonate pathway from statin use has led to several theories investigating their benefit in cancer treatment and prevention (Nielsen, Nordestgaard, & Bojesen, 2012). Statins appear to possess a variety of pleiotropic effects, including inhibition of cell proliferation; enhanced apoptosis; and modulation of inflammation, endothelial function, and angiogenesis (Lochhead & Chan, 2013). A significant body of evidence suggests that statins might have a role in cancer chemoprevention and treatment through their interaction with essential cellular functions (Lochhead & Chan, 2013; Hindler, Cleeland, River, & Collard, 2006). Both in vitro and in vivo studies have demonstrated that statins inhibit tumor growth and induce apoptosis in a variety of tumor cells, including melanoma, glioma, neuroblastoma, and leukemia cell lines (Hindler et al., 2006).

However, there is also contradictory evidence that statins could increase the risk of cancer (Wang et al., 2013). Statins are selectively localized to the liver after ingestion, and less than 5% of a given dose reaches the systemic circulation. Thereby, the usefulness of statins as chemopreventive agents for the majority of cancers is doubted, given their selective hepatic uptake and low systemic availability. The purpose of this article is to review the evidence that supports or refutes the benefits of statins as chemoprotectants for various cancers.

## ANTITUMOR EFFECTS OF STATINS

Cancer can spread throughout the body by proliferation, which makes it challenging to treat. A reduction in the availability of cholesterol could theoretically lead to decreased proliferation and migration of cancer cells through alterations in cell signaling and decreased expression of adhesion molecules. Statins have been linked to the halting of cell-cycle progression in cancer cells resulting in antiproliferative effects, to the inhibition of key cellular functions, and to the increased radiosensitization of cancer cells (Nielsen et al., 2012; Wang et al., 2013).

Many of the downstream products that are inhibited by blocking HMG-CoA are used in cell proliferation because they are required for critical cellular functions such as maintenance of membrane integrity, signaling, protein synthesis, and cell-cycle progression (Nielsen et al., 2012). Disruptions of these processes in malignant cells result in the inhibition of cancer growth and metastasis. In particular, the mevalonate pathway is upregulated by mutated p53 (tumor suppressor protein), which is a common mutation in cancer pathogenesis. Accordingly, inhibition of this pathway by statins reverts the malignancy phenotype of p53-mutated cancer cells (refer to Figure). The decrease in downstream products of this pathway has been linked to apoptosis and reduced matrix-metalloproteinase production and angiogenesis, as well as reduction in the invasiveness of in situ carcinomas.

Statins have been shown to increase regulatory T-cell numbers and functionality in vivo (Wang et al., 2013). Both lipophilic and hydrophilic statins decrease natural killer cell (regulatory cell) cytotoxicity. The immunosuppressive effects of statins might impair host antitumor immune responses, suggesting an opposing effect on tumor development, which should be considered. Lipophilic statins cross the blood-brain barrier more readily (Liu et al., 2014). It has been hypothesized that the preventive effect of statins against cancer may be more apparent with lipophilic than hydrophilic statins due to greater lipid solubility and membrane permeability (refer to Table 1).

**Table 1:**
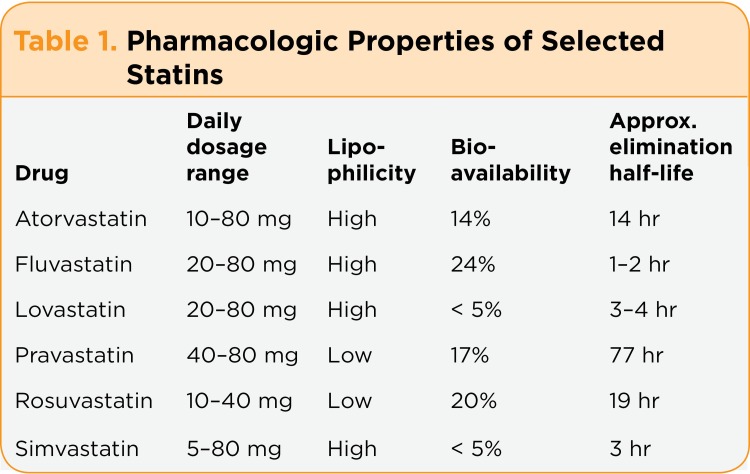
Pharmacologic Properties of Selected Statins

Several laboratory studies evaluating the potential role of statins in cancer have been conducted. Experimental studies have found that statins may induce apoptosis as well as inhibit tumor growth, angiogenesis, and metastasis (Wang et al., 2013). These mechanisms have led to the evaluation of the possible benefit of statins in the prevention and treatment of malignancies.

## CANCER PREVENTION AND TREATMENT

There are mixed results regarding the role of statins in the prevention of cancer. Statins undoubtedly have a role in prevention of cardiovascular complications, and they continue to be explored for malignancy prevention in a variety of tumor types. See Table 2 for a summary of several trials investigating this concept.

**Table 2:**
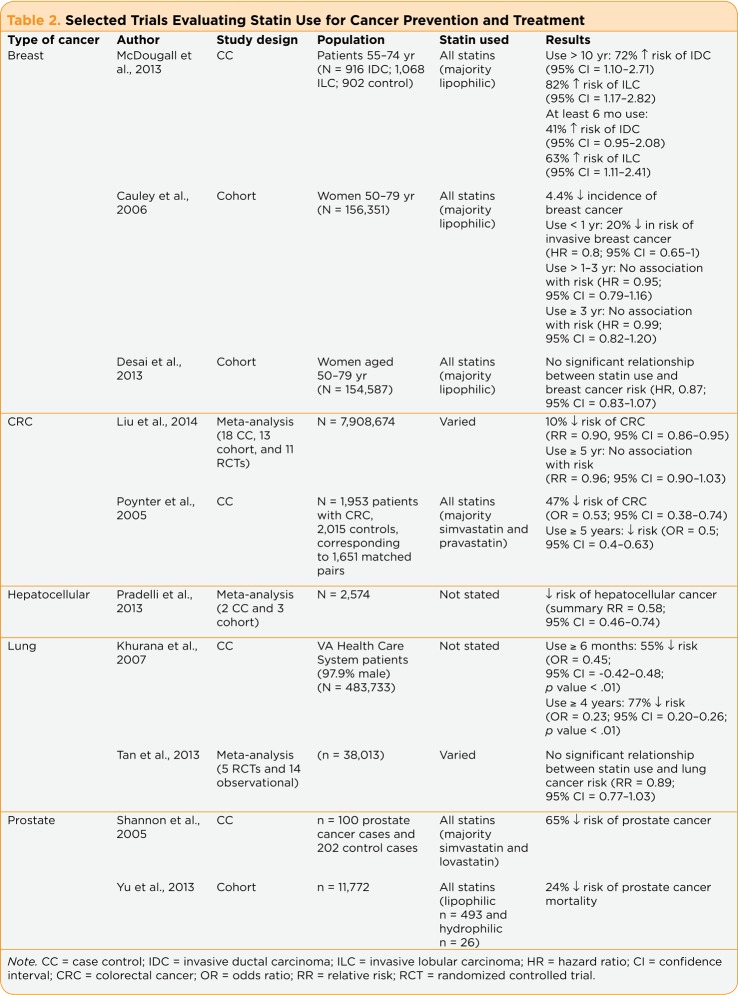
Selected Trials Evaluating Statin Use for Cancer Prevention and Treatment

**Breast Cancer**

Statins have displayed the ability to inhibit the proliferation of breast cancer cells in vitro and in rodents, which led to the interest in whether the use of statins might decrease a woman’s risk of developing breast cancer. However, clinical studies determining the role of statins in breast cancer development have shown mixed results, ranging from a harmful effect to a protective effect, as well as others showing no impact (Desai et al., 2013).

The negative association of statin use in breast cancer has been highlighted by the following trials. One study evaluated long-term statin use in women aged 55 to 74 and found it to be associated with increased risks of both invasive ductal carcinoma (IDC) and invasive lobular carcinoma (ILC; McDougall et al., 2013). In this case-control study, 916 women with IDC and 1,068 women with ILC were compared with 902 women used as a control group. The control group was formed by the Mitofsky-Waksberg method of randomly dialing landline home telephone numbers. Approximately 77% of women in each group had never used statins. Among the 23% of women who used statins for at least 6 months, lipophilic statins were used in 88% of them; the two most commonly prescribed statins were atorvastatin and simvastatin. Women who had taken statin therapy for 10 years or longer had a 72% higher increased risk of IDC and an 82% higher increased risk of ILC compared with women who had never used statins. In women who used statins for at least 6 months, the increased risk of IDC or ILC was more modest (41% and 63%, respectively).

The largest study conducted evaluating statin impact on breast cancer risk supported the notion that statins may have a protective effect against breast cancer (Cauley et al., 2006). This study included 156,351 postmenopausal women, with statins used by 11,710 women (7.5%) of the cohort, including 8,106 (69.2% of statin users) who used a lipophilic statin. Over an average follow-up of 6.7 years, 4,383 invasive breast cancers were confirmed by medical records. Breast cancer incidence was approximately 4.4% lower among women using statins, with breast cancer incidences of 4.09 per 1,000 person-years among statin users and 4.28 per 1,000 person-years among nonusers. Statin use of less than 1 year resulted in a 20% reduction in invasive breast cancer. Statin use for more than 1 year was not associated with the risk of developing breast cancer. The study determined that there was an 18% lower incidence of breast cancer with the use of lipophilic statins in postmenopausal women (p = .02).

Desai and colleagues collected information on statin use at baseline and at years 1, 3, 6, and 9 in 154,587 postmenopausal women aged 50 to 79 who were participants in the large observational or clinical trial arms of Women’s Health Initiative study (Desai et al., 2013). Statins were used by 11,584 (7.5%) of the women at baseline, with the majority of statins (69.5%) used being lipophilic. After 9 years, the annualized rate of breast cancer was 0.42% among statin users and 0.42% among nonusers. The multivariable-adjusted model showed no significant relationship between statin use and breast cancer risk (hazard ratio [HR] = 0.87; 95% confidence interval [CI] = 0.83–1.07). In this study, there was no significant reduction in breast cancer risk due to lipophilic statin use. Statin use for less than 1 year’s duration was associated with a trend toward an inverse association of breast cancer development (HR = 0.78; 95% CI = 0.63–0.98). There was also no significant trend by overall duration of statin use. In the women who developed breast carcinoma, there was no effect of statins on tumor stage, grade, or hormone receptor status. Overall, in this analysis, statins were not associated with an increased or decreased breast cancer risk.

**Colorectal Cancer**

There is a long-standing debate about whether statins have chemopreventive properties against the development of colorectal cancer (CRC), and the results remain inconclusive (Liu et al., 2014). Thus, Liu et al. completed a meta-analysis of 42 studies (18 case control studies, 13 cohort studies, and 11 randomized controlled trials) with a total of 7,908,674 patients that examined the chemopreventive properties of statins against CRC. Overall, statin use was associated with a 10% reduction in the risk of CRC (relative risk [RR] = 0.90; 95% CI = 0.86–0.95). Thirteen studies that were evaluated included colon and rectal cancers separately. Their results when evaluated separately revealed a reduction in the risk of rectal cancer (RR = 0.81; 95% CI = 0.66–0.99; p = .040) but not in the risk of colon cancer (RR = 0.97; 95% CI = 0.92–1.01; p = .142). Long-term statin use (≥ 5 years) did not significantly affect the risk of CRC (RR = 0.96; 95% CI = 0.90–1.03). When the type of statin used was evaluated, the association between lipophilic statin use and lower CRC risk was significant (RR = 0.88; 95% CI = 0.85–0.93; p < .001); however, with the use of hydrophilic statins, the risk was not significantly increased or decreased (RR = 0.88; 95% CI = 0.76–1.02; p = .088).

The Molecular Epidemiology of CRC study also evaluated statins in CRC (Poynter et al., 2005). It included 1,953 patients with CRC and 2,015 controls, corresponding to 1,651 matched pairs. Simvastatin and pravastatin were the two most commonly used statins (55.6% and 41.5%, respectively). In the patients with long-term statin use (≥ 5 years), there was a significantly reduced relative risk of CRC in comparison to nonusers of statins. The use of statin therapy was associated with a 47% relative reduction in the risk of CRC after adjustment for other known risk factors such as aspirin use, presence of physical activity, hypercholesterolemia, family history of CRC, and ethnic group. The results from this trial indicated that there is a strong inverse association between the risk of CRC and the long-term use of statins. Both of the available reports evaluating statin use in CRC demonstrated that there is an inverse association between the risk of CRC and statin use.

**Hepatocellular Cancer**

In addition to trials in breast cancer and CRC, there are studies evaluating the utility of statins for prevention of hepatocellular carcinoma. The meta-analysis conducted by Pradelli and colleagues suggests that statin use was inversely related to the risk for hepatocellular cancer, with an over 40% decrease in liver cancer risk among statin users, regardless of the duration of statin exposure (Pradelli et al., 2013). This meta-analysis included five observational studies (two case-control and three cohort studies) and comprised a total of 2,574 cases of hepatocellular cancer. Patients who had statin treatment compared with no treatment experienced decreased rates of hepatocellular cancer. This meta-analysis suggests a favorable effect of statins on hepatocellular cancer risk without regard to a duration-risk relationship.

**Lung Cancer**

Khurana et al. conducted a retrospective case control cohort study to determine if statins have a protective effect against the development of lung cancer in 483,733 patients enrolled in the Veterans Affairs Health Care System (Khurana, Bejjanki, Caldito, & Owens, 2007). This population consisted of veterans with 97.9% of the participants being male. Statin use occurred in 1,994 of the 7,280 patients (27.4%) with lung cancer and in 161,668 of 476,453 patients (33.9%) without lung cancer. Statin use for more than 6 months was associated with a 55% risk reduction of lung cancer. There was a 77% reduction in the odds for developing lung cancer among those who used statins for 4 or more years. The protective effect of statins was seen across all ages, ethnic groups, body mass indexes, smoking history, alcohol use, and comorbidity of diabetes.

A meta-analysis conducted by Tan et al. included 19 studies (5 randomized controlled studies and 14 observational studies) with 38,013 lung cancer patients (Tan et al., 2013). There was no evidence of an association between statin use and risk of lung cancer among randomized controlled trials (RR = 0.91; 95% CI = 0.76–1.09), cohort studies (RR = 0.94; 95% CI = 0.82–1.07), or case-control studies (RR = 0.82; 95% CI = 0.57–1.16). This article concluded there was no association between statin use and the risk of developing lung cancer, regardless of the type of trial assessed.

A second meta-analysis of 20 studies (5 randomized controlled trials, 8 cohort studies, and 7 case-control studies) evaluating patients with lung cancer found that statin use did not significantly affect the risk of lung cancer when statins were taken at daily doses for cardiovascular event prevention (RR = 0.89; 95% CI = 0.78–1.02; Wang et al., 2013). A total of 4,980,009 participants, including 37,650 lung cancer cases, were involved.

To determine if patient gender had an impact on lung cancer risk, the following studies were conducted. Cheng and colleagues investigated the association between statin use and lung cancer risk in female patients and found that statin use was not associated with the risk of lung cancer development in females (Cheng, Chiu, Ho, & Yang, 2012). Another study by Hippisley-Cox and Coupland investigated statin use and lung cancer risk among male and female populations independently. The results revealed that statin use was not associated with lung cancer risk in either female (odds ratio [OR] = 1.00; 95% CI = 0.81–1.23) or male patients (OR = 1.05; 95% CI = 0.97–1.13; Hippisley-Cox & Coupland, 2010).

**Prostate Cancer**

Prostate cancer is one of the leading malignancies affecting men. A case control study by Shannon and colleagues included 100 patients with prostate cancer and 202 controls for the analysis of prostate cancer risk (Shannon et al., 2005). In this study the use of statin therapy, particularly lovastatin and simvastatin, was significantly greater among controls (49%). The use of lovastatin and simvastatin was due to formulary medication restrictions at the Veterans Affairs Medical Center during the evaluated time. Men with any recorded statin use had a 65% lower risk of prostate cancer compared with nonusers. When the data were stratified by Gleason score, the percentage of reduction in risk of less aggressive (Gleason score 6) disease was not significant, but the percentage of reduction in risk of more aggressive (Gleason score ≥ 7) disease was at 76%.

Yu and colleagues conducted a cohort analysis of 11,772 men newly diagnosed with early or locally advanced prostate cancer to determine whether the use of statins after prostate cancer diagnosis is associated with a decreased risk of cancer-related mortality (Yu et al., 2014). The use of statins was associated with a 24% decreased risk of prostate cancer mortality as recorded in death certificates and all-cause mortality. The decreased risks of prostate cancer mortality were greater in patients who had also used statins prior to diagnosis. A 23% decreased risk of developing prostate cancer was observed with lipophilic statins, and a 35% decreased risk was observed with hydrophilic statins.

## CONCLUSION

Most of the data observing statin use in relation to cancer prevention and treatment have been observational and retrospective. There are no large, prospective trials published in the literature to determine the exact role statins have with regard to cancer. In breast cancer and CRC, it is suggested that there might be a difference in the benefit of statins based on the lipophilic properties of the agent used. Another aspect to be investigated is the difference in dose and duration of statin use in cancer prevention. At this time, no single statin agent and dose has been recommended for cancer prevention in the general population.

In addition, further research should be conducted to determine if the use of statins decreases the severity of cancer. For example, statin users were almost as likely as nonusers to have CRC diagnosed at an early stage of disease (stage I or II) as compared with a later stage (stage III or IV; OR = 0.90; 95% CI = 0.54–1.50; p = .69; Poynter et al., 2005). Colorectal cancer was somewhat less likely to be poorly differentiated among statin users (6.4%) than among nonusers (8.6%), although this difference was not significant (p = .48). Large, prospective, randomized studies are needed in order to answer the question of whether statins can be used to prevent and/or treat various cancers.
